# The Incontinence Praying Ability and the Incontinence Quality of Life Questionnaires for Muslim Women: A Confirmatory Study of the Malay Language Versions

**DOI:** 10.21315/mjms2018.25.5.11

**Published:** 2018-10-30

**Authors:** Kueh Yee Cheng, Dariah Mohd Yusoff, Hanis Ismail, Nyi Nyi Naing

**Affiliations:** 1 Unit of Biostatistics and Research Methodology, School of Medical Sciences, Universiti Sains Malaysia, 16150 Kubang Kerian, Kelantan, Malaysia; 2 School of Health Sciences, Universiti Sains Malaysia, 16150 Kubang Kerian, Kelantan, Malaysia; 3 Institute for Community (Health) Development (i-CODE), Universiti Sultan Zainal Abidin, 21300 Kuala Nerus, Terengganu, Malaysia

**Keywords:** validity, reliability, confirmatory factor analysis, praying ability, incontinence, quality of life

## Abstract

**Background:**

The aim of the present study was to determine the validity and reliability of the Malay versions of the Incontinence Praying Ability (I-PA) and the Incontinence Quality of Life (I-QoL) questionnaires among childbearing-aged Muslim women.

**Methods:**

This cross-sectional study included 338 childbearing-aged Muslim women (mean age: 31.1 years; SD = 5.57) who attended clinics at Hospital Universiti Sains Malaysia. Confirmatory factor analysis was conducted to determine the construct validity of the Malay versions of the I-PA and the I-QoL, while composite reliability was used to examine their internal consistency reliability.

**Results:**

The hypothesised models of the I-PA and the I-QoL consisted of 10 items with one latent variable and 22 items with three latent variables, respectively. The hypothesised models of the I-PA and the I-QoL did not have good fit. Modifications included co-varying the residual terms for items within the subscales, which resulted in improved fit indices for the I-PA (CFI = 0.976 TLI = 0.961, RMSEA = 0.068) and the I-QoL (CFI = 0.948, TLI = 0.938, RMSEA = 0.063). The composite reliability of the I-PA was 0.93, and the I-QoL ranged from 0.91 to 0.94.

**Conclusion:**

The Malay versions of the I-PA and I-QoL were considered to be valid, reliable questionnaires measuring incontinence QoL among Muslim women of childbearing age.

## Introduction

Many women experience uncontrollable or involuntary leakage of urine, and urinary incontinence (UI) is recognised as common, debilitating and costly condition for women (1, 2). UI is prevalent among women but is preventable, curable and manageable (3). Common factors associated with UI among women include age, childbearing, body mass index, previous hysterectomy and other comorbidities (4). Pregnant women, postnatal women and menopause women have higher UI rates. Previous research reported that childbearing women face risk factors for UI, and the main contributing factor is damage to important muscle tissue and nerves caused by vaginal delivery (5, 6). Rortveit et al. also reported that pregnancy can lead to UI caused by mechanical changes, hormonal changes or both (5).

According to Broome, UI affects around 15%–35% of the adult ambulatory population, and its impacts include depression, self-efficacy and poor quality of life (QoL) (7). Depression has also been suggested to be associated with UI (8, 9). Previous studies have found that UI leads to anxiety and hysteria (10, 11). Other studies have reported associations of UI with social isolation, loneliness, falls, nursing home admission, hospitalization and increased need for informal care, especially among older women (12–15). In addition, women with UI are more likely to report lower QoL and self-efficacy because the effect of self-efficacy on QoL is positively related to each other (16, 17). Measuring the QoL of people with UI, therefore, is crucial in medical and health care.

Several questionnaires assess QoL among people with UI, but most do not consider QoL related to the ability to pray, which is a spiritual practice for Muslims. Muslims with UI, especially women with severe UI, are more likely to face difficulty performing *salah* or praying (18). Prayer requires women to stand, bend and sit several times, and maintaining cleanliness while performing these prayers is difficult for women with UI as these movements may cause leakage (19). This situation may cause feelings of insecurity as women repeat ritual cleansing before successfully completing their prayers for fear that leakage will prevent them from meeting their religious obligations (18, 19). Performing prayer is part of the daily routine of Muslims in Malaysia, so assessing the impact of UI on their ability to perform prayers is important.

A study on Egyptian women indicated that the inability to perform prayers while experiencing UI problems was the most distressing QoL issue (20). Experiencing UI problem has much more distressing effects on Muslim women than Jewish, Hindu or Christian women (19, 21). Moreover, an urogynaecologist stated that few Muslim women had been assessed for UI (19). This situation has resulted from the lack of a validated instrument to measure the effects of UI on QoL among Muslim women and a lack of knowledge about sensitive conditions and perceptions among Muslim women. For instance, Wilkinson found that Asian Muslim women felt that health professionals were not interested in their problems and did not provide adequate support (22).

Due to the lack of instruments assessing QoL among Muslims with UI, Dariah et al. developed and validated a questionnaire measuring the impacts of UI on QoL related to praying ability (18, 23). Dariah also translated the Incontinence Quality of Life (I-QoL), a well-established questionnaire measuring QoL among people with UI (18). The present study was aimed at examining the validity and reliability of the Malay versions of the Incontinence Praying Ability (I-PA) and I-QoL for measuring the impact of UI on QoL and praying ability among childbearing-aged Muslim women.

## Materials and Methods

### Study Design, Participants and Data Collection

The participants in this cross-sectional study were women who attended Hospital Universiti Sains Malaysia (USM) obstetrics and gynaecology, outpatient, specialist and staff clinics. The inclusion criteria were: childbearing women ages 18–45 years old who were Muslim, experienced UI and attended particular clinics at Hospital USM for treatment, follow-up treatment or medical check-up. Data were collected from January to March 2016. The self-administered questionnaire pack included demographic form, Malay versions of the I-PA and the I-QoL was distributed to the eligible participants, and the researchers briefed the participants on the study, the procedures and the confidentiality of responses. Informed consent was obtained from all individual participants before they completed the questionnaire, which took 15–20 min. This study was approved by the USM Human Research Ethics Committee [JEPeM Code: USMKK/PPP/JEPeM/[265.4 (1.6)].

### Measures

The demographic form included questions on the type of clinic the participants visited and their gender, age, race, education level, employment status, household income and size, and number of children.

The I-PA scale in Malay language was constructed and developed by Dariah in 2011 by adapting the design of I-QoL, as has been reported in detail (18). The initial validation on the Malay version of the I-PA using exploratory factor analysis was conducted by Dariah et al. (23). The I-PA measured the impact of UI on QoL related to praying ability and consisted of 10 items within the domain of praying ability, a spiritual practice for Muslim. The items were scored on a five-point scale: 1 = extremely, 2 = quite a bit, 3 = moderately, 4 = a little, 5 = not at all. The I-PA showed good internal consistency reliability with Cronbach’s alpha of 0.94 (23). All 10 items were included as one factor measuring the impact of UI on QoL related to praying ability among Muslims.

The I-QoL, an established questionnaire measuring QoL among people with UI (24), was translated into Malay using standard forward and backward translation, as described by Dariah (18). The I-QoL consisted of 22 items and three subscales (factors). The items were scored on a 5-point Likert scale: 1 = extremely, 2 = quite a bit, 3 = moderately, 4 = a little, 5 = not at all. The factors in the I-QoL were avoidance and limiting behaviour (ALB), psychosocial impacts (PI) and social embarrassment (SE). The I-QoL showed good internal consistency reliability, with Cronbach’s alpha for each factor of 0.91–0.96 and test-retest stability with intra-class correlations of 0.72–0.97 in a cross-cultural study conducted by Bushnell et al. (25).

### Data Analysis

Data analysis was conducted using Mplus 7.3 (26). Data were screened for missing values, normality and outliers before confirmatory factor analysis (CFA) was performed. The assumption of multivariate normality was checked using Mardia’s multivariate skewness and kurtosis test with *P*-values less than 0.05. In the present study, the assumption of multivariate normality was not met, so estimator MLM (Satorra-Bentler chi-square), which was robust to non-normality, was used in CFA.

The initial hypothesised measurement models of the I-PA and the I-QoL were developed and tested in CFA. The initial hypothesised measurement model for the I-PA consisted of one latent variable (I-PA subscale) and 10 observed variables (I-PA items). The initial hypothesised measurement model for the I-QoL consisted of three latent variables (I-QoL subscales) and 22 observed variables (I-QoL items). The factor loadings of the models were examined, and 0.50 was used as the cut-off point to identify problematic items (27). The modification index was used as a guide to add covariance among the items’ residuals. The models were re-specified (removing items and adding covariance on the residuals) after the researchers ensured adequate theoretical support. Several fit indices were used to determine the best fit measurement model for the I-PA and the I-QoL. The fit indices and the recommended fit values were: the comparative fit index (CFI) and Tucker and Lewis index (TLI), with a desired value of more than 0.950; root mean square error of approximation (RMSEA), with a desired value of less than 0.070; and standardised root mean square (SRMR), with a desired value of less than 0.080 (27, 28). After the best fit measurement model was identified, composite reliability (CR) for CFA was applied to measure the reliability of the items within the factors (29). The recommended CR value was 0.70 or more (27). Further analysis was conducted to support the validity of the I-PA by examining the Pearson correlation between the I-PA and the I-QoL subscales. The I-PA was hypothesised to be significantly correlated with the I-QoL subscales.

#### Sample Size

The sample size needed for CFA depended on the complexity of a measurement model (27). Hair et al. suggested that if the communalities were low or the model included multiple under-identified constructs (i.e. fewer than three items), then a minimum sample size of 300 was required to test a measurement model of CFA (27). In this study, although the I-PA and I-QoL constructs consisted of more than three items, low communalities on some items could be expected. Therefore, we proposed that a minimum sample size of 300 was required for the present study.

## Results

### Participants’ Characteristics

A total of 338 childbearing Muslim women completed the questionnaire. All the participants were married and had experienced pregnancy and UI at some point in their lives. The descriptive statistics of the participants’ characteristics are shown in Table 1

### Descriptive Statistics of the I-PA and I-QoL

Table 2 presents the frequency and percentage of the respondents for each item in the I-PA and the I-QoL based on a 5-point Likert scale. The mean and standard deviation for each item in the I-PA and the I-QoL are also presented. The full set of items in the I-PA and the I-QoL is listed in the Appendix.

### Measurement Model of I-PA

The hypothesised measurement model for the I-PA, which consisted of one factor with 10 items, did not have good fit with the data based on several fit indices (CFI = 0.879; TLI = 0.844; RMSEA (90% CI) = 0.137 (0.122, 0.153); SRMR = 0.056). All the factor loadings were more than the recommended cut-off point of 0.50. Re-specification of the measurement model was carried out as suggested by the modification index in the CFA results. The modification of the initial model included adding covariance between the residuals for items P2 and P1, P3 and P1, P3 and P2, P4 and P2, P4 and P3, P10 and P8, and P10 and P9. Adding covariance between these items’ errors seemed reasonable as these items indicated the impact of UI on praying ability. The final measurement model of the I-PA was established with acceptable fit indices (CFI = 0.976; TLI = 0.961; RMSEA (90% CI) = 0.068 (0.049, 0.088); SRMR = 0.029). The factor loadings of the initial and final measurement models of the I-PA are presented in Table 3. Based on the final measurement model, CR was computed to be 0.93 (95% CI = 0.91, 0.94), indicating good construct reliability.

### Measurement Model of the I-QoL

The hypothesised measurement model for the I-QoL, which consisted of three factors with 22 items, did not have good fit with the data based on several fit indices (CFI = 0.891; TLI = 0.878; RMSEA (90% CI) = 0.088 (0.081, 0.095); SRMR = 0.046). However, all the factor loadings were higher than the recommended cut-off point of 0.50. The model was re-specified to improve the fit indices. We examined the modification index in the CFA results that suggested that adding covariance between the residuals of some items would improve the model fit indices. After adequate theoretical support was carried out, we decided to add covariance between the residuals for some items within the factors. Covariance was iteratively added between the residuals of some items in the model: Q1 and Q2, Q2 and Q3, Q5 and Q6, Q5 and Q7, Q6 and Q7, Q8 and Q14, Q11 and Q13, Q12 and Q19, Q15 and Q16, Q15 and Q17, and Q16 and Q17. The resulting model fit the data well (CFI = 0.948; TLI = 0.938; RMSEA (90% CI) = 0.063 (0.055, 0.070); SRMR = 0.037). Although the CFI and TLI values were not higher than 0.95, Hair et al. suggested that CFI and TLI values more than 0.920 were acceptable in models with more than 12 observed variables (items) and more than 250 sample size (27). The factor loadings of the initial and final measurement models of the I-QoL are presented in Table 4. Based on the final measurement model, the CR (95% CI) for ALB, PI and SE was 0.91 (0.90, 0.93), 0.94 (0.93, 0.95) and 0.93 (0.92, 0.95), respectively, indicating good construct reliability.

### Correlation of the I-PA and I-QoL

The I-PA and the three factors of I-QoL were found to be strongly correlated, with values of 0.64–0.71 (see Table 5). This further supported the validity of the I-PA construct for measuring QoL related to praying ability for people with UI.

## Discussion

The purpose of this study was to confirm the validity of the measurement models of the Malay versions of two questionnaires measuring praying ability and QoL among Muslim women who experienced UI. Prayer and other spiritual acts are important practices among Muslims, and Muslims who have UI symptoms may feel discomfort when performing prayers, which might negatively affect their QoL (18). The majority of questionnaires measuring QoL among people with UI do not address the component of spirituality, particularly the act of praying among Muslims. This study, therefore, makes an important contribution: a validated, reliable scale measuring the impact of UI on QoL related to praying ability. This scale could complement the I-QoL in measuring the level of QoL among Muslims with UI.

The development of the I-PA is an important step to determining individual QoL related to praying ability among Muslims. In this study, we conducted a confirmatory examination of the one-factor structure of the I-PA. Among the questionnaires measuring the QoL of people with UI, the I-PA is the first measure specifically developed to address the impact on QoL from UI experienced by Muslims during prayer. The I-PA items were first developed in English and then translated into Malay (18). The Malay version of the I-PA was validated with a study population from whom Malay was the most commonly spoken and well-understood language. The study results confirmed that the one-factor structure with the 10 I-PA items and the reliability of the I-PA were satisfactory. These findings were in line with our previous exploratory study, finding the I-PA construct to be valid and reliable based on exploratory factor analysis and Cronbach’s alpha (23).

The present study confirmed the validity of the Malay version of I-QoL with three factors and 22 items. CFA results showed that the Malay version of the I-QoL measurement model was fit based on several fit indices. Furthermore, the CR was satisfactory for each I-QoL subscale. Developing measurement tools for the Malay version of the I-QoL is crucial as there is no valid Malay-language questionnaire to measure the QoL of people with UI symptom. The English version of the I-QoL has been widely used by researchers to measure QoL among people with UI and has been translated and validated in more than 16 languages (25, 30–32). However, there has been no validation study on the I-QoL in Malay using a confirmatory approach. This study thus provides an important resource for researchers, physicians and health educators interested in evaluating the level of QoL among people with UI symptoms: a validated Malay version of the I-QoL based on a confirmatory study.

Based on the study findings, we propose that the I-PA and the I-QoL should be used to evaluate the QoL of Muslims experiencing UI symptoms. The praying ability measurement in the I-PA was highly correlated with all the I-QoL subscales (ALB, PI and SE). This result was expected as the I-PA was developed based on the I-QoL framework specifically measuring QoL for people with UI symptoms. The present study fills the research gap on specific measurements of QoL among observant Muslims with UI symptoms.

The present study has some limitations. First, the data were collected at a single hospital, which could limit the generalisability of the findings to other hospitals and states in Malaysia. Second, the self-administrated survey could be subject to measurement and response bias, potentially reducing the accuracy of the survey data. Third, the study participants constituted a specific group of people: muslim women of childbearing age. Future research could include wider socio-demographic populations, such as people who are obese or overweight, people with other comorbidities and older people who are more likely to have UI symptoms.

## Conclusion

The findings showed that the Malay version of the I-PA with 10 items and the I-QoL with three factors and 22 items were valid and reliable among the 338 Muslim childbearing-aged women. Both questionnaires are recommended for use in future research examining QoL among Muslims with UI. The additional scale of I-PA on I-QoL will enable more comprehensive evaluation of QoL among Muslims experiencing UI.

## Figures and Tables

**Table 1:** Descriptive statistics of sociodemographic characteristics (*n* = 338)

Variables	*n* (%)	Mean (SD)
Clinic		
Obstetrics and gynecology	210 (62.1)	
Outpatient	68 (20.1)	
Specialist	30 (8.9)	
Staff	30 (8.9)	
Age in year		31.1 (5.57)
Race		
Malay	336 (99.4)	
Others	2 (0.6)	
No. of children		2.0 (2.00) *
Educational level		
Primary school	11 (3.3)	
Secondary school	154 (45.6)	
Diploma	75 (22.2)	
Bachelor	83 (24.6)	
Master	15 (4.4)	
Employment status		
Employed	168 (49.7)	
Housewife	167 (49.4)	
Student	3 (0.9)	
Household income		
Less than RM1,000	69 (20.4)	
RM1,000 until RM2,000	97 (28.7)	
RM2,000 until RM3,000	58 (17.2)	
RM3,000 until RM4,000	51 (15.1)	
More than RM4,000	63 (18.6)	
No. of family member		4.0 (2.00) *

Note:

* Median and interquartile range were reported due to data skewed

**Table 2:** Descriptive statistics of items for the I-PA and the I-QoL

Item	Five rating likert scale, *n* (%)					Mean (SD)

	Extremely	Quite a bit	Moderately	A little	Not at all	
I-PA:						
P1	32 (9.5)	65 (19.2)	46 (13.6)	83 (24.6)	112 (33.1)	3.53 (1.37)
P2	36 (10.7)	57 (16.9)	51 (15.1)	91 (26.9)	103 (30.5)	3.50 (1.36)
P3	18 (5.3)	32 (9.5)	59 (17.5)	91 (26.9)	138 (40.8)	3.88 (1.20)
P4	15 (4.4)	28 (8.3)	49 (14.5)	78 (23.1)	168 (49.7)	4.05 (1.17)
P5	13 (3.8)	25 (7.4)	46 (13.6)	69 (20.4)	185 (54.7)	4.15 (1.14)
P6	15 (4.4)	29 (8.6)	44 (13.0)	65 (19.2)	185 (54.7)	4.11 (1.19)
P7	11 (3.3)	17 (5.0)	48 (14.2)	70 (20.7)	192 (56.8)	4.23 (1.07)
P8	24 (7.1)	34 (10.1)	45 (13.3)	59 (17.5)	176 (52.1)	3.97 (1.30)
P9	12 (3.6)	14 (4.1)	29 (8.6)	56 (16.6)	227 (67.2)	4.40 (1.04)
P10	13 (3.8)	25 (7.4)	42 (12.4)	68 (20.1)	190 (56.2)	4.17 (1.14)
I-QoL:						
ALB						
Q1	18 (5.3)	45 (13.3)	82 (24.3)	111 (32.8)	82 (24.3)	3.57 (1.15)
Q2	15 (4.4)	50 (14.8)	75 (22.2)	106 (31.4)	92 (27.2)	3.62 (1.16)
Q3	7 (2.1)	28 (8.3)	58 (17.2)	93 (27.5)	152 (45.0)	4.05 (1.07)
Q4	17 (5.0)	45 (13.3)	66 (19.5)	101 (29.9)	109 (32.2)	3.71 (1.19)
Q10	15 (4.4)	49 (14.5)	83 (24.6)	106 (31.4)	85 (25.1)	3.58 (1.14)
Q11	17 (5.0)	32 (9.5)	62 (18.3)	92 (27.2)	135 (39.9)	3.88 (1.18)
Q13	19 (5.6)	30 (8.9)	64 (18.9)	93 (27.5)	132 (39.1)	3.86 (1.19)
Q20	11 (3.3)	39 (11.5)	58 (17.2)	107 (31.7)	123 (36.4)	3.86 (1.13)
PI						
Q5	8 (2.4)	33 (9.8)	67 (19.8)	81 (24.0)	149 (44.1)	3.98 (1.12)
Q6	8 (2.4)	32 (9.5)	56 (16.6)	68 (20.1)	174 (51.5)	4.09 (1.13)
Q7	7 (2.1)	27 (8.0)	53 (15.7)	75 (22.2)	176 (52.1)	4.14 (1.08)
Q9	7 (2.1)	26 (7.7)	55 (16.3)	76 (22.5)	174 (51.5)	4.14 (1.08)
Q15	9 (2.7)	29 (8.6)	45 (13.3)	82 (24.3)	173 (51.2)	4.13 (1.10)
Q16	6 (1.8)	25 (7.4)	49 (14.5)	76 (22.5)	182 (53.8)	4.19 (1.05)
Q17	9 (2.7)	23 (6.8)	39 (11.5)	80 (23.7)	187 (55.3)	4.22 (1.07)
Q21	5 (1.5)	23 (6.8)	48 (14.2)	79 (23.4)	183 (54.1)	4.22 (1.02)
Q22	8 (2.4)	21 (6.2)	47 (13.9)	75 (22.2)	187 (55.3)	4.22 (1.05)
SE						
Q8	16 (4.7)	35 (10.4)	48 (14.2)	81 (24.0)	158 (46.7)	3.98 (1.21)
Q12	34 (10.1)	42 (12.4)	63 (18.6)	88 (26.0)	111 (32.8)	3.59 (1.33)
Q14	6 (1.8)	30 (8.9)	39 (11.5)	86 (25.4)	177 (52.4)	4.18 (1.06)
Q18	16 (4.7)	44 (13.0)	64 (18.9)	98 (29.0)	116 (34.3)	3.75 (1.19)
Q19	12 (3.6)	24 (7.1)	56 (16.6)	105 (31.1)	141 (41.7)	4.00 (1.09)

**Table 3:** Standardised factor loading of initial and final measurement model of the I-PA

Items	Initial model		Final model	
	Factor loading	Residual variance	Factor loading	Residual variance
P1	0.81	0.34	0.77	0.41
P2	0.81	0.34	0.76	0.42
P3	0.81	0.34	0.77	0.41
P4	0.90	0.19	0.89	0.21
P5	0.85	0.27	0.87	0.25
P6	0.90	0.20	0.91	0.17
P7	0.89	0.20	0.91	0.18
P8	0.82	0.34	0.81	0.35
P9	0.74	0.45	0.75	0.43
P10	0.80	0.36	0.80	0.37

P = I-PA’s items

**Table 4:** Standardised factor loading of initial and final measurement model of the I-QoL

Factor	Items	Initial model		Final model	
	
		Factor loading	Residual variance	Factor loading	Residual variance
ALB	Q1	0.75	0.44	0.73	0.47
	Q2	0.74	0.45	0.72	0.48
	Q3	0.82	0.32	0.81	0.34
	Q4	0.81	0.34	0.81	0.35
	Q10	0.81	0.35	0.80	0.35
	Q11	0.85	0.27	0.85	0.28
	Q13	0.78	0.39	0.78	0.40
	Q20	0.80	0.36	0.81	0.34
PI	Q5	0.84	0.30	0.84	0.30
	Q6	0.87	0.24	0.86	0.27
	Q7	0.89	0.21	0.87	0.25
	Q9	0.84	0.29	0.85	0.29
	Q15	0.90	0.18	0.88	0.23
	Q16	0.89	0.20	0.86	0.26
	Q17	0.91	0.18	0.88	0.23
	Q21	0.85	0.28	0.86	0.26
	Q22	0.81	0.34	0.82	0.32
SE	Q8	0.88	0.23	0.87	0.24
	Q12	0.81	0.34	0.83	0.31
	Q14	0.88	0.22	0.86	0.25
	Q18	0.83	0.32	0.84	0.30
	Q19	0.88	0.23	0.89	0.21

Q = I-QoL item-number

**Table 5:** Correlation between the I-PA and the I-QOL’s subscales

Factors	Mean (SD)	I-PA	*P*-value
ALB	3.77 (0.95)	0.82	< 0.001
PI	4.15 (0.95)	0.83	< 0.001
SE	3.90 (1.04)	0.85	< 0.001
I-PA	4.00 (1.02)	-	-

## Appendix

The English and Malay version of I-PA were presented in section (i). The original English version of I-QoL developed by Patrick et al was presented in section (ii). All the items in Malay language indicated as in italic.

(i) Incontinence Praying Ability (I-PA) instrument

1 = extremely (*sangat banyak*), 2 = quite a bit *(banyak),* 3 = moderately (*sederhana*), 4 = a little (*sedikit*), 5 = not at all (*langsung tiada kesan*)

**Table:**

No. The impact of urine leakage on your life	1	2	3	4	5
1. I worried whether I am really clean each time of praying. *Saya merasa risau sama ada saya sudah betul-betul bersih setiap kali hendak mengerjakan solat.*					
2. I have to change my sarong/pants at each praying time. *Saya perlu menukar kain/seluar yang saya pakai setiap kali bersolat.*					
3. I have to take a bath and change my clothes for each praying time. *Saya akan mandi tiap kali sebelum mengerjakan solat.*					
4. I repeat my ablution for several time before praying. *Saya mengulangi wuduk saya beberapa kali sebelum solat.*					
5. I keep on praying even though I know that I could not maintain my ablution because of my incontinence. *Saya terus bersolat walaupun saya sedar saya akan terkencing lagi pada bila-bila masa.*					
6. I could not concentrate (*kusyuk*) on praying because of my incontinence. *Saya tidak kusyuk semasa bersolat kerana masalah tidak boleh mengawal kencing saya ini.*					
7. I use to repeat praying for several times because of my incontinence. *Saya mengulangi solat saya beberapa kali kerana masalah tidak boleh mengawal kencing saya ini.*					
8. I feel like I am sinful if I keep on praying when I am not ritually clean/smelly. *Saya merasa berdosa kerana terus mengerjakan solat walaupun dalam keadaan yang kurang bersih/berbau.*					
9. I always skip praying because of my incontinence. *Saya kerap meninggalkan solat kerana masalah tidak boleh mengawal kencing saya ini.*					
10. I feel like God did not accept my praying. *Saya merasa solat saya seakan tidak diterima oleh Tuhan.*					

Note: The reference for English and Malay version of I-PA: Dariah MY. Post-natal urinary incontinence in Kelantan, Malaysia: a mixed methods study. PhD diss. Adelaide: Flinders University, Adelaide, Australia; 2011

(i) Incontinence Quality of Life (I-QOL) instrument

1 = extremely (*sangat banyak*), 2 = quite a bit *(banyak),* 3 = moderately (*sederhana*), 4 = a little (*sedikit*), 5 = not at all (*langsung tiada kesan*)

**Table:**

No. The impact of urine leakage on your life	1	2	3	4	5
1. I worry about not being able to get to the toilet on time. *Saya bimbang sekiranya saya tidak sempat ke tandas tepat pada masanya.*					
2. I worry about coughing and sneezing. *Saya bimbang untuk batuk atau bersin.*					
3. I have to be careful about standing up after sitting down. *Saya mesti berhati-hati ketika hendak berdiri selepas duduk.*					
4. I worry where the toilets are in new places. *Saya bimbang memikirkan di mana letaknya tandas ketika saya berada di tempat baharu.*					
5. I feel depressed. *Saya rasa tertekan (risau).*					
6. I don’t feel free to leave my home for long periods of time. *Saya rasa tidak bebas meninggalkan rumah untuk tempoh yang panjang.*					
7. I feel frustrated because my UI prevents me from doing what I want. *Saya kecewa kerana masalah tidak boleh mengawal kencing menghalang saya melakukan perkara yang saya suka.*					
8. I worry about others smelling urine on me. *Saya bimbang orang lain akan terhidu bau air kencing pada badan saya.*					
9. Incontinence is always on my mind. *Masalah tidak boleh mengawal kencing saya ini sentiasa bermain dalam fikiran saya.*					
10. It’s important for me to make frequent trips to the toilet. *Penting bagi saya pergi ke tandas lebih kerap.*					
11. Because of my incontinence, it is important to plan every detail in advance. *Disebabkan masalah tidak boleh mengawal kencing ini, penting bagi saya untuk merancang setiap aktiviti dengan lebih awal.*					
12. I worry about my incontinence getting worse as I grow older. *Saya bimbang jika masalah tidak boleh mengawal kencing saya ini, menjadi lebih teruk apabila umur saya semakin meningkat.*					
13. I have a hard time getting a good night’s sleep. *Saya sukar mendapatkan tidur malam yang mencukupi.*					
14. I worry about being embarrassed or humiliated be cause of my incontinence. *Saya bimbang dimalukan atau dihina kerana masalah tidak boleh mengawal kencing saya ini.*					
15. My incontinence makes me feel like I’m not a healthy person. *Masalah tidak boleh mengawal kencing saya ini menyebabkan saya merasa tidak sihat.*					
16. My UI makes me feel helpless. *Masalah tidak boleh mengawal kencing saya ini menyebabkan saya merasa tidak berdaya.*					
17. I get less enjoyment out of life because of my UI. *Saya kurang menikmati kehidupan kerana masalah tidak boleh mengawal kencing saya ini.*					
18. I worry about wetting myself. *Saya sentiasa bimbang akan terkencing dan membasahi seluar/kain saya.*					
19. I feel like I have no control over my bladder. *Saya rasa seperti tidak dapat mengawal pundi kencing saya.*					
20. I have to watch what I drink. *Saya perlu menjaga jenis dan jumlah air yang saya minum.*					
21. My UI limits my choice of clothing. *Masalah tidak boleh mengawal kencing saya ini menyekat jenis baju yang boleh saya pakai.*					
22. I worry about having sex. *Saya bimbang mengadakan hubungan kelamin (seks).*					

Note: The reference for English version of I-QoL: Patrick DL, Martin ML, Bushnell DM, Yalcin I, Wagner TH, Buesching DP. Quality of life of women with urinary incontinence: further development of the incontinence quality of life instrument (I-QOL). *Urology*. 1999;**53(1)**:71–76.
